# Scleral fixation of iris-Intraocular lens complex (Reper®) with Canabrava double-flanged technique: a case report

**DOI:** 10.5935/0004-2749.20230060

**Published:** 2023

**Authors:** Altan Atakan Özcan, Aynura Sarıyeva Aydamırov

**Affiliations:** 1 Department of Ophthalmology, School of Medicine, Cukurova University, Adana, Turkey.; 2 Department of Ophthalmology, Adana City Training and Research Hospital, Adana, Turkey.

**Keywords:** Aphakia, Aniridia, Lens implantation, intraocular lenses, Scleral surgery, Visual acuity, Humans, Case reports, Afacia/etiologia, Aniridia, Implante de lente intrao­cular, Lentes intraoculares, Esclera/cirurgia, Acuidade visual, Humanos, Relatos de casos

## Abstract

A 38-year-old patient who developed aphakia and aniridia secondary to trauma suffered from vision loss. To improve her vision, an iris-intraocular lens complex (Reper^®^) was fixed to the sclera with Canabrava’s double-flanged technique. There was a satisfactory increase in the patient’s visual acuity and no complications were observed during the 6-months follow-up. Canabrava technique simplifies and improves the fixation of the iris-intraocular lens complex to the sclera. It is a safe option that does not require scleral flaps or knots.

## Abstract (pt)

Uma paciente de 38 anos desenvolveu afacia e ani­ridia secundárias a um trauma, levando à perda da visão. Para melhorar sua visão, um complexo de íris e lente intraocular (Reper^®^) foi fixado à esclera com a técnica de flange duplo de Canabrava. Houve um aumento satisfatório na acuidade visual do paciente e nenhuma complicação foi observada durante o acompanhamento de 6 meses. A técnica de Canabrava simplifica e melhora a fixação do complexo de íris e lente intraocular na esclera. É uma opção segura que não requer retalhos ou pontos esclerais.

## INTRODUCTION

In the correction of aphakia, the anterior chamber, iris, ciliary sulcus, or sclera can be used to place the intraocular lens (IOL)^(1,2)^. Sutured and sutureless techniques have been developed for scleral fixation^(2)^. Custom-made iris prostheses and iris-IOL complexes are important options for the treatment of acquired or congenital iris defects in aphakia. They are specially designed according to the particular iris color and the condition of the patient’s lens^(3)^. Therefore, this case report demonstrates implantation of the Reper iris-IOL complex using the Canabrava double-flanged technique, which is worth considering as it provides such advantages as low costs, gradual learning curve, and no need for scleral flaps or knots, in the patient with aphakia and concurrent aniridia.

## CASE REPORT

**Figure 1:**
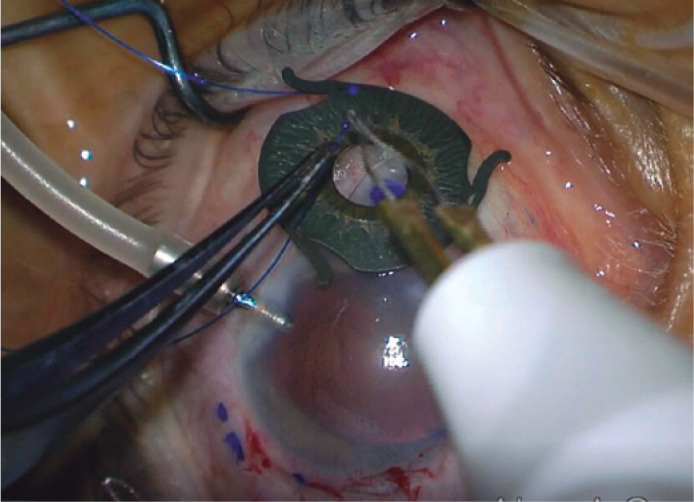
The 5-0 polypropylene suture end is passed through the eyelet of a Reper iris-IOL complex and heated by the thermocautery to create the flange.

**Figure 2:**
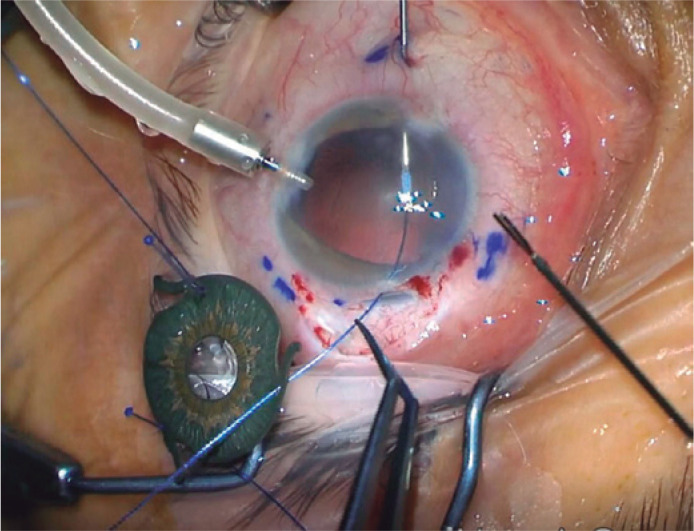
By the help of 25G microforceps, 5-0 polypropylene suture is pushed into the 27 gauge needle’s inner cavity. The needle is then used as an external guide for the suture end within the sclera.

**Figure 3:**
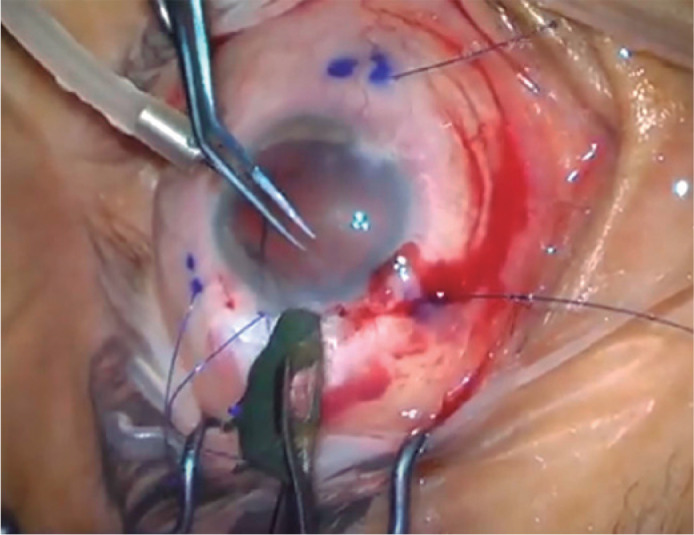
The iris-IOL complex is folded and introduced into the anterior chamber by the help of lens introducer.

**Figure 4:**
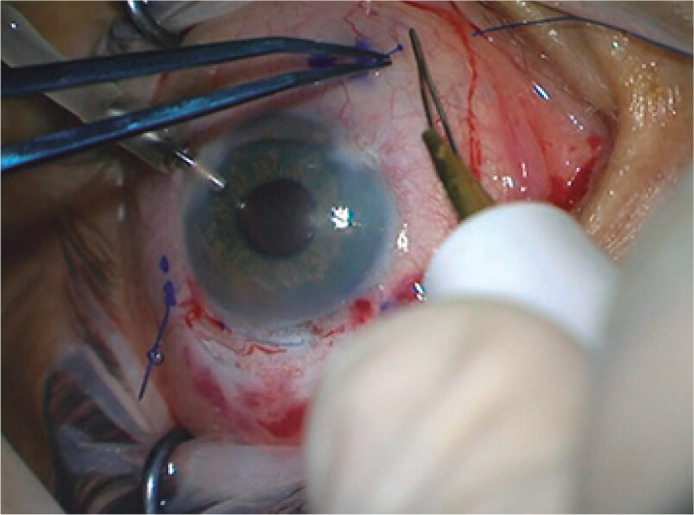
After IOL positioning, the sutures are cut 2 mm from their base and heated to form the second flanges.

A 38-year-old female patient complained of vision loss and a cosmetic problem in her left eye. She had a penetrating trauma when she was 4 years old. Corneal haze, aniridia, aphakia, and exotropia were observed, and corrected distance visual acuity was 2/10 in her left eye and central corneal thickness was 582 µM. There was no apparent pathology on the fundus examination. Scleral fixation of the artificial iris-IOL complex (Reper^®^, Model C) was planned to the left eye using the Canabarava Double-Flanged Technique. This artificial iris-IOL complex has a 3.5-mm pupillary aperture with a 13-mm overall diameter and is made from a hydrophobic acrylic material^(4)^. The iris color that will match the color of the patient’s other eye was selected from the color match catalog. At the start of the operation, the sclerotomy sides were marked on the conjunctiva 2 mm apart from the limbus. The intrascleral length of the tunnel was about 2 mm. A beveled and long intrascleral tunnel is important to avoid the risk of endophthalmitis and is a very critical point to minimize the risk of decentration or tilt^(5)^. To fixate this foldable iris-IOL complex by using the double-flanged technique, we needed to perform a 5.2-mm posteriolimbal incision and a self-sealing tunnel using a crescent knife. To prevent hypotony anterior chamber maintainer is introduced. The 5-0 polypropylene suture end was passed through the first eyelet of a Reper iris-IOL complex and heated by the thermocautery to create the first flange (Figure 1). This was repeated for the other 2 eyelets. Then, as an external guide, a 27 gauge hypodermic needle was used to perform transconjunctival sclerectomy. This needle entered the anterior chamber and by the help of forceps and 25G microforceps the other part of the suture was pushed into the inner cavity of the needle (Figure 2). The suture was withdrawn from the sclerotomy side and this was repeated with other sutures of the eyelets. The iris-IOL complex was folded and introduced into the anterior chamber (Figure 3). The complex was centered by pulling the suture ends externalized 2 mm from the limbus at each side. The sutures were cut 2 mm from their base and heated to the last final second flanges (Figure 4), which would be inserted into the scleral tunnel. After the operation, the uncorrected distance visual acuity of the left eye increased to 5/10 at the 6-month postoperative period and the intraocular pressure was 14 mmHg.

## DISCUSSION

In 1991, Lewis described a classic sutured scleral fixation technique^(6)^, but suture knots may erode, causing conjunctival damage. To solve this problem, the sutures are covered with scleral flaps. IOL dislocation due to suture breakage has been described with the use of 10-0 polyprolene suture^(7)^. Although studies using 9-0 polyprolene and 7-0 Gore-Tex sutures that are thicker than 10-0 polyprolen seem successful, the long-term results are yet unknown^(8)^. Therefore, a search for new techniques has been started to overcome suture-related problems. In this regard, glued IOL technique of Agarwal et al. came for the first time^(9)^. Yamane et al. described a sutureless technique^(10)^. The most important disadvantages of this method are that it requires a learning curve, haptic manipulation is difficult, and it is necessary to make sure that haptics are embedded in the scleral tunnel. Another flapless and sutureless scleral fixation technique named double-flanged technique was described by Canabrava et al.^(11,12)^. They used the double-flanged technique to fixate IOL to the sclera with 5-0 polyprolene, which is thicker than 10-0 polyprolene and more resistant to abrasions and breakages^(12)^.Canabrava et al. reported conjunctival hyperemia and damage around the flange in one patient at 1 month postoperatively because of improper insertion^(5)^. Roditi et al. reported endophthalmitis in a patient 6 months postoperatively after scleral fixation with the Canabrava technique^(13)^. They reported that the cause of endophthalmitis was the exposed polypropylene flange. Therefore, long-term follow-up for conjunctiva and flange stabilization is recommended.

Aphakia may be accompanied by partial iris defects or aniridia. There are different options for aniridia or iris defects management in patients with aphakia. Custom-made iris protheses (ArtificialIris, Customflex, HumanOptics) with three-piece IOL implantation or iris-IOL complex (Reper) can be used, as we used in our patient^(3)^.

In conclusion, scleral fixation with the Canabrava technique is a safe option that can be applied in patients with aphakia and concurrent aniridia. The main issue is the insertion of the flanged 5-0 polypropylene suture into the sclera instead of direct burying. Canabrava technique has a low cost and gradual learning curve, which does not require scleral flaps or knots.

According to our knowledge, this is the first case presentation in the literature where the Reper iris-IOL complex was implanted using the Canabrava double-flanged technique. However, because it is a new technique, further studies with more patients and longer follow-ups are necessary to assess the exact outcomes, including potential risks of endophthalmitis and conjunctival erosion.
